# Nonwoven/Nanomembrane Composite Functional Sweat Pads

**DOI:** 10.3390/membranes12121230

**Published:** 2022-12-05

**Authors:** Muhammad Bilal Qadir, Mohammed Jalalah, Muhammad Usman Shoukat, Adnan Ahmad, Zubair Khaliq, Ahsan Nazir, Muhammad Naveed Anjum, Abdul Rahman, Muhammad Qamar Khan, Rizwan Tahir, M. Faisal, Mabkhoot Alsaiari, Muhammad Irfan, Saeed A. Alsareii, Farid A. Harraz

**Affiliations:** 1 Department of Textile Engineering, National Textile University, Faisalabad 37610, Pakistan; 2 Promising Centre for Sensors and Electronic Devices (PCSED), Advanced Materials and Nano-Research Centre, Najran University, Najran 11001, Saudi Arabia; 3 Electrical Engineering Department, College of Engineering, Najran University, Najran 61441, Saudi Arabia; 4 Department of Materials, National Textile University, Faisalabad 37610, Pakistan; 5 Department of Applied Chemistry, Government College University, Faisalabad 38000, Pakistan; 6 Department of Textile & Clothing, Karachi Campus, National Textile University, Karachi 74900, Pakistan; 7 Department of Chemistry, Faculty of Science and Arts, Najran University, Najran 11001, Saudi Arabia; 8 Department of Chemistry, Faculty of Science and Arts at Sharurah, Najran University, Najran 11001, Saudi Arabia; 9 Department of Surgery, College of Medicine, Najran University, Najran 11001, Saudi Arabia

**Keywords:** antimicrobial, sweat pads, nonwoven web, super absorbent, electrospun nanomembranes, moisture management, comfort

## Abstract

Sweat is a natural body excretion produced by skin glands, and the body cools itself by releasing salty sweat. Wetness in the underarms and feet for long durations causes itchiness and an unpleasant smell. Skin-friendly reusable sweat pads could be used to absorb sweat. Transportation of moisture and functionality is the current challenge that many researchers are working on. This study aims to develop a functional and breathable sweat pad with antimicrobial and quick drying performance. Three layered functional sweat pads (FSP) are prepared in which the inner layer is made of an optimized needle-punched coolmax/polypropylene nonwoven blend. This layer is then dipped in antimicrobial ZnO solution (2, 4, and 6 wt.%), and super absorbent polymer (SAP) is embedded, and this is called a functional nonwoven (FNW1) sheet. Electrospun nanofiber-based nanomembranes of polyamide-6 are optimized for bead-free fibers. They are used as a middle layer to enhance the pad’s functionality, and the third layer is again made of needle-punched optimized coolmax/polypropylene nonwoven sheets. A simple nonwoven-based sweat pad (SSP) is also prepared for comparison purposes. Nonwoven sheets are optimized based on better comfort properties, including air/water vapor permeability and moisture management (MMT). Nonwoven webs having a higher proportion of coolmax show better air permeability and moisture transfer from the inner to the outer layer. Antimicrobial activity of the functional nonwoven layer showed 8 mm of bacterial growth, but SSP and FSP showed only 6 mm of growth against Staphylococcus aureus. FSP showed superior comfort and antibacterial properties. This study could be a footstone toward highly functional sweat pads with remarkable comfort properties.

## 1. Introduction

Sweating is a normal and natural human body activity and is also related to body cleaning. Although the amount of sweat produced varies from person to person, it is always in a hot and humid climate [1,2]. Sweat in the underarms causes discomfort as our primary garment gets moist. Owing to the moisture level, sweat causes plain clothes to become soggy in the underarm area. Itching and a foul odor occur when the underarm is wet for an extended period. Most humans also experience discomfort during sweating [3,4]. In addition, sweating causes garment staining, resulting in the garment’s lousy appearance. This issue also results in other complications, like various garment designs, such as tight-fitting garments and using artificial materials in garments [5,6].

Furthermore, another issue related to sweating is a nasty odor. The odor may result from several sources [7]. The smell and the detection of sweating underarms are awkward for people, especially those who tend to sweat heavily. When people face this problem [8], deodorants or antiperspirants are applied to the skin. Lynne M. B. Blank et al. introduced a way that reduced underarm sweating and odor by using a cross-linked microporous copolymer containing methacrylate and acrylate units [9]. The fatty acid components of sweat are absorbed in polymers and reduce the access of skin bacteria by the acid, which reduces body odor. However, deodorants and scented powders are not appropriately effective for many people. Moreover, these products are investigated as skin irritants [10]. Sweat pads reabsorb the discharged liquids, avoiding the strain on garments and providing comfort to the wearer by enhancing the dryness of the skin surface where sweating occurs [11].

Varied materials are used in distinctive designs of sweat pads, and the ultimate function of these pads is to absorb sweat, minimize odor, and prevent clothes from staining [12,13]. Various antimicrobial agents are used to diffuse the smell. Wicking materials are used to transfer the liquid from one layer to another layer [14]. The first layer, next to the skin, is nonwoven and made of a material with wicking properties, e.g., nylon, polyester, and rayon [15]. Different fibers and polymers are used as absorbent materials. Materials with high absorbing capacity can be used in long-lasting sweat pads [16]. For this purpose, in most research, nonwovens are made from different fibers bonded together by various methods, such as chemical or mechanical. The processing techniques are spun-bond, melt-blown, water jet, and needle-punched [17]. Nonwovens are high-tech engineered fabrics with many applications, including absorbent hygiene products [18]. Nonwoven [19] plays an essential role in modern life due to innovative and versatile high-performance products that give specific functions, including absorbency, softness, strength, and bacterial barrier [20]. Nonwoven fabric forming technology is more budget-friendly than knitted and woven. Needle punching technology is used by most pad manufacturers [21].

The main focus of sweat pads is to absorb perspiration and control the malodorous compound inside the places during their utilization as antiperspirants [22]. There are various disclosures in the art, especially in sanitary napkins and absorbing pads, which describe unconventional materials that provide malodorous control and absorbing capacity [23]. However, various problems are faced with this type of absorbing pad [24]. These pads perform well when people have light to moderate sweating conditions but fail when the sweating condition is heavy [25]. Most of the pads are also not breathable, so more sweat can be produced, causing discomfort to the user. Pads in which a breathable sheet is used deal with micro-level, which wet the underarm portion of the user’s garment. In functional sweat pads, antimicrobial agents [26] are used along with the absorption of sweat [27]. These agents reduce the number of malodorous compounds that are produced by sweating. This invention relates to sweat-absorbing pads, having an absorbent core, a back layer, and malodors’ control systems [28]. The presence of the absorbent core with the malodor control system provides an incredible improvement by minimizing underarm smell and absorbing sweat [2,29]. The nanoweb can control breathability and moisture management at the optimum level because of its nano-sized porosity.

There are limited studies on the development of sweat pads, whereas optimization of the nonwoven structures for the development of sweat pads is missing. Moreover, a study on developing a sweat pad with a nanofiber layer is missing in the literature. Electrospun nanofiber-based composites have numerous applications, such as biomedical applications [30], drug delivery [31], water/air treatment [32], photocatalysts [33,34], microsensor [35], and stretchable thermoelectrics [32] owing to their unique characteristics including, large surface to volume ratio, excellent absorbency, good electric conductivity, and moisture management. Plasmonic nanofiber membrane, having excellent water vapor transmission, was successfully reported as interfacial solar vapor generators [36]. The presence of nanofiber layers improves the moisture transportation and performance of the sweat pad. It was also previously reported that PA6 nanofiber with a finer diameter shows higher water uptake compared to nanofiber with a coarser diameter [37]. Similarly, another study of blended nanofiber membranes also revealed that nanofiber webs, composed of nanofiber having finer diameters, showed an increase in water absorption [38]. The smaller diameter attributed to the large pore size resulted in higher moisture absorption. There is no study to fabricate functional sweat pads with antimicrobial and anti-perspiration performance and a breathable structure using electrospun nanomembranes. The functional sweat pads containing a three layered structure will be manufactured through nonwoven and electrospinning. The inner layer of coolmax, polypropylene, and their blend will transport sweat from the inner to the middle absorbing layer. The super absorbent material will be incorporated into the first nonwoven layer with a coating of antimicrobial ZnO nanoparticles to minimize the odor near the underarm portion. Polyamide 6 (PA 6) nanomembrane is used as a second layer of the functional sweat pads to enhance breathability and vaporization of sweat. An optimized first nonwoven layer will be used as a third layer, which works as a protective layer.

## 2. Materials and Methods

Coolmax fiber (multi-channeled structure) was purchased from LYCRA^®^ United State of America (Wilmington, DE, USA). Polypropylene fiber (used for better moisture transfer) was purchased from Ningbo LY Industry Co., Ningbo, China. The SAP (super absorbent material) was purchased from Landy Enterprise Ltd., Hefei, China, and zinc oxide nanoparticles (antimicrobial agents), acetic acid, and polyamide 6 (PA-6) from Sigma Aldrich, St. Louis, MO, USA.

### 2.1. Development of A Functional Sweat Pad

**Figure 1:**
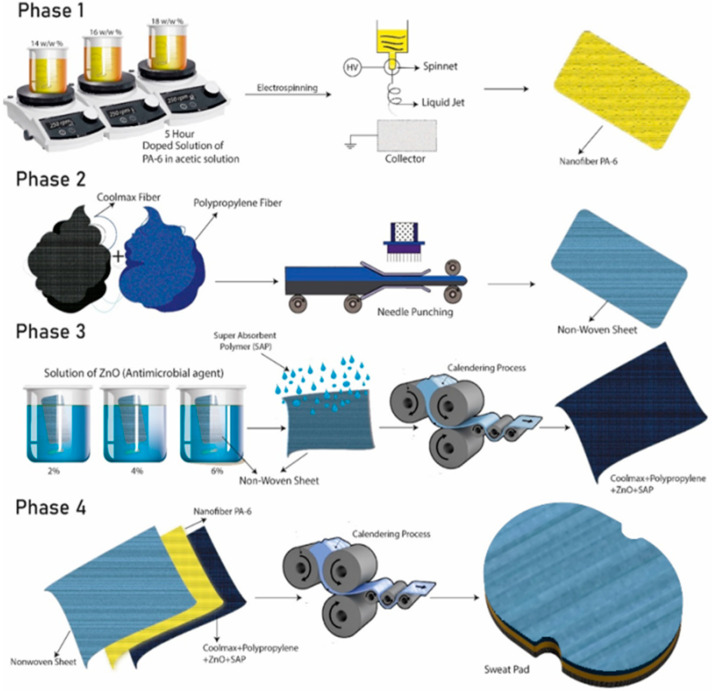
Sequence of phases and fabrication of functional sweat pads.

The development of functional sweat pads was divided into four phases. All the phases are summarized in Figure 1. In phase I, the electrospinning solution and process parameters were optimized to get bead-free nanofibers. A detailed experimentation plan is given in Table 1. PA-6 solutions were prepared using acetic acid solvent (14, 16, and 18 wt.%) under magnetic stirring (250 rpm) at room temperature. PA-6 solutions were electrospun at 150, 150, 200, and 250 µL/h flow rates; and 28, 30, and 32 kV voltage. An optimized and bead-free sample of nanofibers was used as a middle layer in a sweat pad to increase the moisture transfer from the inner layer to the outer layer of nonwovens. The optimized PA-6 nanofiber membrane was selected after analyzing the diameter of the nanofiber, and sample NF12 was selected as an optimized sample for the middle nanofiber membrane. The comfort characteristics of the NF12 are given in Table 2.

In Phase II, the nonwoven webs were developed using coolmax and polypropylene fibers with different blend ratios (0–100%). These webs were manufactured on a needle punch nonwoven setup (Dong-Won Roll (DW-N/P, Incheon, South Korea)). These nonwoven webs were characterized by their comfort properties, and an optimized nonwoven web was selected for the next phase. Sample # 1 for the nonwoven layer was selected as an optimized sample, as highlighted (NW1) in Table 3, and its characteristics are given in Table 4.

In phase III, the ZnO nanoparticles disperse solution was prepared by sonication of ZnO nanoparticles in water for 3 h using a bath sonicator. Then NW1 was dipped in ZnO solution (for antimicrobial properties) at various concentrations (2, 4, and 6 wt.%). Then super absorbent polymers (SAP) were embedded on this nonwoven web through sprinkling and calendering. The melting temperature of coolmax and polypropylene is 255 °C and 160 °C, respectively. The temperature of the calendering roller was kept at 100 °C for the incorporation of SAP on the first nonwoven layer. The optimized functional nonwoven sample FNW1 having 6% ZnO nanoparticles coating was selected, and its comfort properties are given in Table 5.

In phase IV, a nanofiber-based functional sweat pad (NFSP) was developed by joining all three layers using the calendering roller. The optimized PA-6 nanofibers sample NF12 obtained in phase I was used as a middle layer. NW1, having 100% coolmax fiber, obtained in Phase II, was used as the third layer (outer), and functional nonwoven web (FNW1), having 100% coolmax and 6% ZnO nanoparticles coating, prepared from phase III, was used as the first layer (inner). A simple two-layer sweat pad (SSP) and a two-layer functional nonwoven sweat pad (FSP) were also fabricated for comparison to the developed NFSP sweat pad.

### 2.2. Characterization and Techniques

The morphology of electrospun PA-6 nanofibers was observed by using a Field-emission scanning electron microscope (FE-SEM) (FEI Nova Nano SEM 450, USA). The samples were coated with gold using a sputter coater and placed on the sample holder. The SEM images were taken from different locations at different magnifications. The Image J software (1.53t, NIH, USA) was used to check the diameter of PA6 nanofibers from other sites, and the average value of the diameter was recorded. Almost 50 readings were taken to calculate the mean diameter of a single sample. Air permeability was determined as per ASTM D737 using the air permeability tester (SDL ATLAS, USA). The pressure was set at 100 Pa with a surface head of 20 cm^2^. The moisture management characteristics of the composite sweat pad were analyzed through the moisture management tester (MMT) through the standard method AATCC TM195. The inner side of the sample faced the drop to measure the moisture transfer from the inner to the outer surface. The thermal resistance of the sweat pad was measured through the sweating guard hot plate using EN ISO 11092:2014. The plate’s temperature was kept at 35 °C, and environmental conditions were set at 20 °C temperature and 65% relative humidity. The water vapor permeability of the nanofiber membrane, nonwoven layer, and composite sweat pad was analyzed through the cup method according to BS 7209 standard. The environmental conditions were kept at 20 °C temperature and 65% relative humidity. The WVP of the sample was measured through equation 1, given below

(1) $$\mathrm{WVP}=\frac{24\times M}{A\times t}$$

where *M* is the amount of water, in grams, that evaporates during the test (the difference between the initial weight of the water and the final weight of the water in a cup after the test), *A* is the cross-sectional area of the cup (m^2^), and *t* is the time duration (h) of the test.

AATCC 147 is a test to detect bacterial activity on fabric or textile materials. The test method determines the antibacterial activity of diffusible antimicrobial agents on treated textile materials. First, bacterial colonies are prepared in an N-Broth solution at 37 °C for 24 h. A dilution of a solution designed in step 1 is then made, preferably by serial dilution. Then N-Agar is sterilized at 121 °C for 15 min, poured into Petri dishes, and given time to solidify. Next, 50 μL diluted inoculums in step 3 are taken, spread on an Agar plate, and the sample is placed in an appropriate shape. The Petri dish is cured and incubated for 24 h at 37 °C. The zone of inhibition for each sample is then measured.

## 3. Results and Discussions

### 3.1. Optimization of Nanofiber Fiber Morphology and Diameter

**Figure 2:**
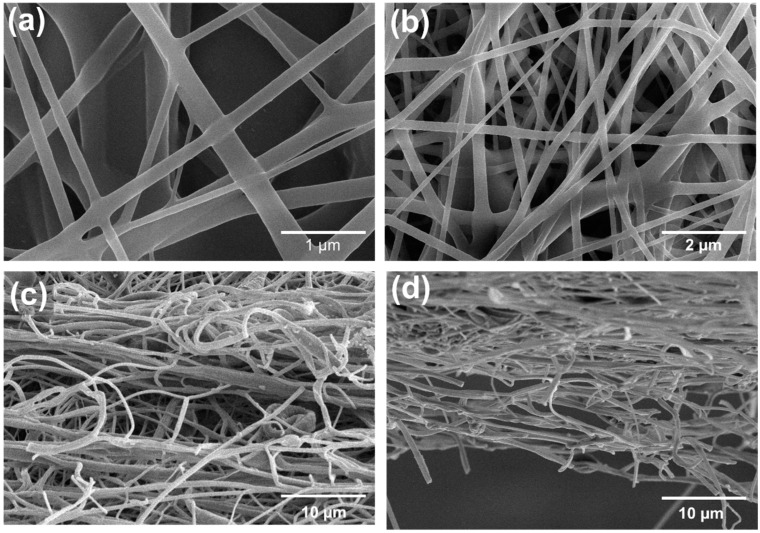
(**a**,**b**) SEM images of PA6 nanofibers at optimized conditions. (**c**,**d**) Cross-sectional view of optimized PA6 nanofiber membrane.

The fiber morphology of the PA6 nanofiber diameter was investigated through the FE-SEM. Figure 2a,b show the SEM images of the nanofiber diameters, and Figure 2c,d show the cross-sectional view of the nanofiber membrane. It can be observed that spun PA nanofibers are bead-free and possess a circular morphology.

**Figure 3:**
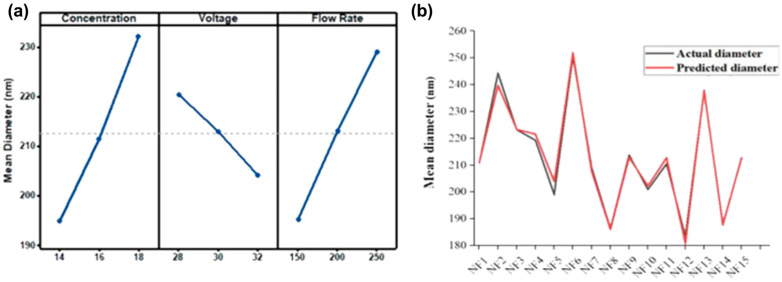
(**a**) Effect of concentration, voltage, and flow rate on mean diameter. (**b**) Validity of the regression model through actual and predicted diameter at optimized conditions 14% PA6 concentration, 30 kV applied voltage, and 150 µL/h flow rate.

Electrospun nanofibers’ mean diameter and morphology depend on solution and processing parameters. The impact of the concentration on the mean diameter was evaluated at 14, 16, and 18 wt.% concentration, at voltages of 28, 30, and 32 kV, and at flow rates of 150, 200, and 250 µL/h of PA6 nanomembrane. It was observed that the mean diameters of electrospun PA 6 nanofibers increased by increasing the concentration of polymer, decreased by increasing the value of applied voltage because of an increase in the electric field strength between the needle tip and the collector, and finer PA6 nanofibers were observed at lower flow rates. The effect of concentration, voltage, and flow rate on mean diameter is shown in Figure 3a. It can be observed that more delicate fibers can be obtained at low polymer concentrations, low flow rates, and high voltages with stable Taylor cones. Figure 3b shows the actual and predicted diameter of nanofibers at the different solution and process parameters.

### 3.2. Regression Model for Phase I Parameters

The R^2^ value provides the percentage of variation in the response (mean diameter) that can be explained by the factors included in the regression equation. The higher R^2^ value indicates the corresponding model’s better data fitting and predictive ability. The regression model equation develops the comparison of actual and predicted diameters. It could be observed that the regression model possesses excellent predictability and is close to actual mean diameters obtained through experimentation at different factor levels. Therefore, this regression model contains a higher R^2^ value of 96.65, as shown in Equation (2).

Mean Diameter = 231.6 + 2.24 (Polymer Concentration) − 4.088 (Applied voltage) − 0.228 (Flow rate) + 0.0355 (Polymer concentration × Flow rate) (2)

R^2^ = 96.65

### 3.3. Optimized PA-6 Nanofibers

The optimized nanofiber web (sample NF12) was obtained at 14% PA6 concentration, 30 kV applied voltage, and 150 µL/h flow rate. The optimized nanofiber web membrane has a nanofiber diameter of 180 ± 29 nm with a narrow fiber diameter distribution.

**Figure 4:**
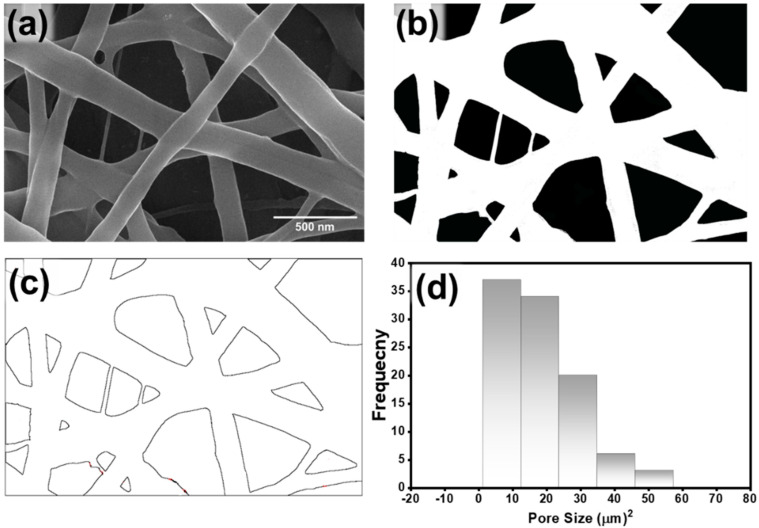
(**a**) Grayscale image, (**b**) Threshold image, (**c**) outlined pore image segmentation of PA6 nanofiber web, (**d**) Pore size distribution of PA6 nanofiber web.

The pore size distribution of the optimized sample NF12 of PA6 nanofiber web is demonstrated in Figure 4a–d. Figure 4a represents the grayscale image of the PA6 nanofiber, which is transferred into binary images as represented in Figure 4b. Then Otsu thresholding is performed to convert the binary image into segmentation. Then, pores are outlined by further processing of the segmented image, as shown in Figure 4c. The optimized sample NF12 exhibits a uniform pore size distribution with an average pore size of 18.04 µm (Figure 4d).

The thermophysiological characteristics such as air permeability, thermal resistance, OMMC, and WVP of the optimized nanofiber web are given in Table 2.

### 3.4. Optimization of Nonwoven Layers through the Evaluation of Comfort Characteristics

The comfort properties, including air permeability, thermal resistance, moisture management, and WVP of all nonwoven layers, are discussed.

**Figure 5:**
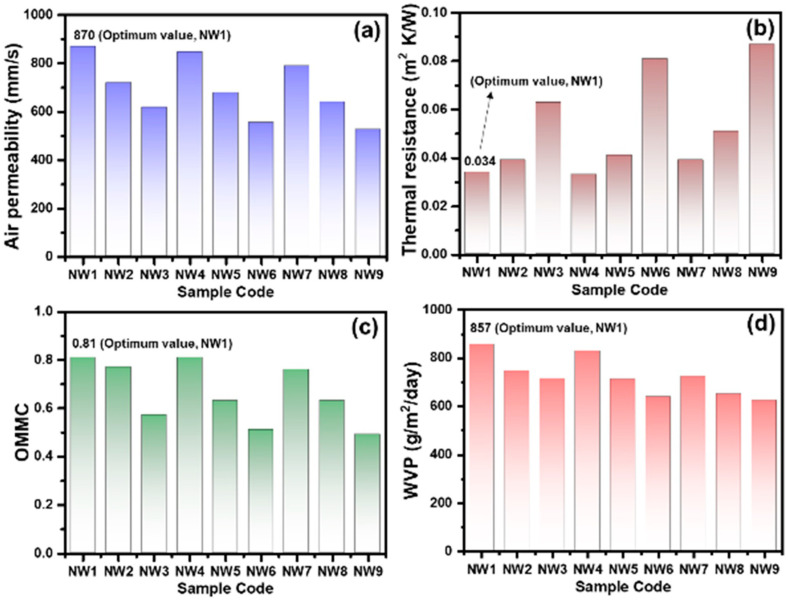
Comfort properties (**a**) Air permeability, (**b**) Thermal resistance, (**c**) OOMC, (**d**) WVP of the nonwoven samples with a different fiber blend ratio.

The air permeability of the nonwoven layers was investigated to evaluate the breathability of the nonwoven layers. The air passing through a material is known as air permeability. Pore characteristics such as size, porosity, and dimension are the parameters that directly influence air permeability. Figure 5a shows that the air permeability of nonwoven samples (NW1–NW9) significantly decreases as the concentration of coolmax decreases. Coolmax fiber has a multichannel structure which helps to pass air easily; therefore, samples with a high concentration of coolmax show air permeability above 800 mm/s.

The thermal resistance of the nonwoven layer estimates the transfer of heat through the nonwoven layers, which as a result, attributes to the comfort of the final product, such as the sweat pad. A sweat guard hot plate is used to measure the thermal resistance of the materials. Figure 5b shows that nonwoven samples NW1–NW9 increase heat resistance by increasing the concentration of polypropylene from 0.033–0.085 m^2^ K/W due to nanopore size, whereas samples with the higher percentage of the coolmax exhibited lower thermal resistance. The sample NW1 having a fiber blend of coolmax and polypropylene (100:0) represents the optimum thermal resistance of 0.033 m^2^ K/W.

The moisture management of the nonwoven layers was evaluated by the overall moisture management (OMMC) of the samples. The OMMC represents the ability of the designed nanofiber-based sweat pad to transfer moisture from the inner surface of the sweat pad to the outer surface (Figure 5c). OMMC is the collective factor of moisture absorption, spreading of moisture, spreading speed, and transfer of moisture from the inner to the outer layer. The nonwoven samples NW1–NW9 show the same trend as air permeability results. Nonwoven samples with a higher percentage of the coolmax fiber show a better OMMC coefficient. The maximum OMMC (0.81) is noted for the sample NW1. Coolmax fiber with a channel cross-section offers a smooth path for moisture flow and transportation. Hence, attributing to the effective moisture management characteristics of the nonwoven layer. The WVP depicts the evaporation of the water through the nonwoven layer under standard atmospheric conditions. The high value of the WVP contributed to the faster drying of the nonwoven layer and offered better breathability. Figure 5d shows the WVP of the different nonwoven layers. The lowest WVP (624 g/m^2^/day) is noted for the nonwoven sample NW9 with a 100% polypropylene blend ratio, whereas sample NW1 (100% coolmax) exhibits the maximum WVP of 857 g/m^2^/day. Coolmax fiber in the nonwoven layer provides the channel path to the water molecules and provides improved evaporation. The comfort properties of the optimized nonwoven sample NW1 are given in Table 4.

### 3.5. Optimization of Functional Layers through the Antimicrobial Activity

**Figure 6:**
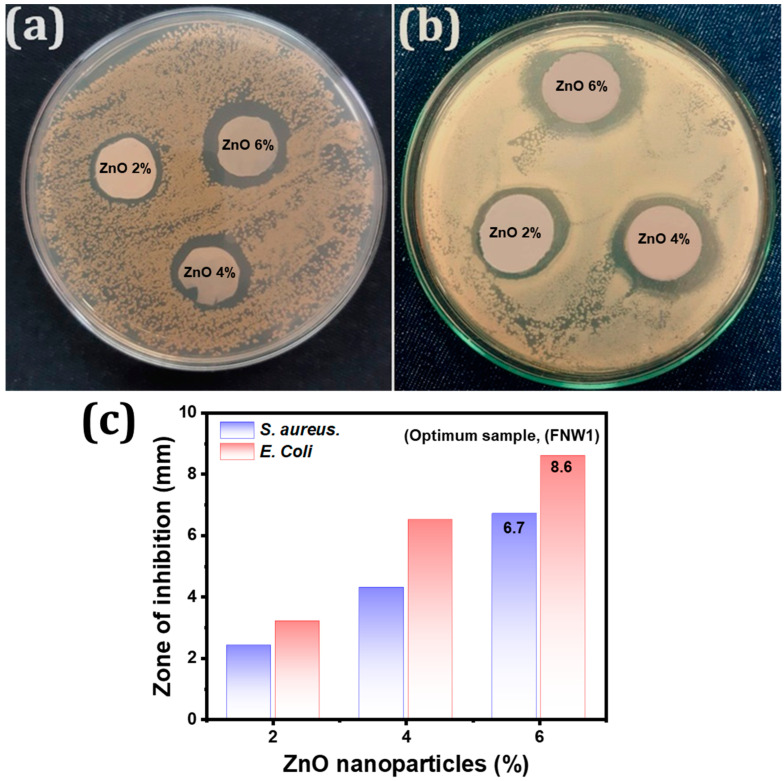
Functional nonwoven layer with different concentrations of ZnO nanoparticles (**a**,**b**) photographic view of antibacterial activity against *S. aureus* and *E. coli* respectively, (**c**) Zone of inhibition.

The disc diffusion method was used to investigate the antimicrobial properties of different samples against *S. aureus* and *E. coli*. The antimicrobial activity and zone of inhibition against *S. aureus* and *E. coli,* respectively, for different concentrations of ZnO (2%, 4%, and 6%), can be seen in Figure 6a–c. All three samples show antimicrobial activity in the presence of bacterial colonies. The functional nonwoven samples’ ZnO concentration of 2%, 4%, and 6% shows inhibition zones of 2.4 mm, 4.3 mm, and 6.7 mm against *S. aureus,* respectively. While the inhibition zone against the *E. coli* is noted as 3.2 mm, 6.5 mm, and 8.6 mm, respectively. The antibacterial activity of the functional nonwoven layer is increased with the high concentration of ZnO nanoparticles in the coating solution. Moreover, it is also observed the ZnO nanoparticles performed more effectively against *E. coli* than *S. aureus*. The functional layers (FNW1), having ZnO nanoparticles of 6% in the solution, exhibited significant antibacterial activity compared to other samples and used an optimized functional nonwoven layer. The comfort properties of the optimized functional nonwoven sample FNW1 are given in Table 5.

### 3.6. Comfort Characteristics of Developed Sweat Pad

**Figure 7:**
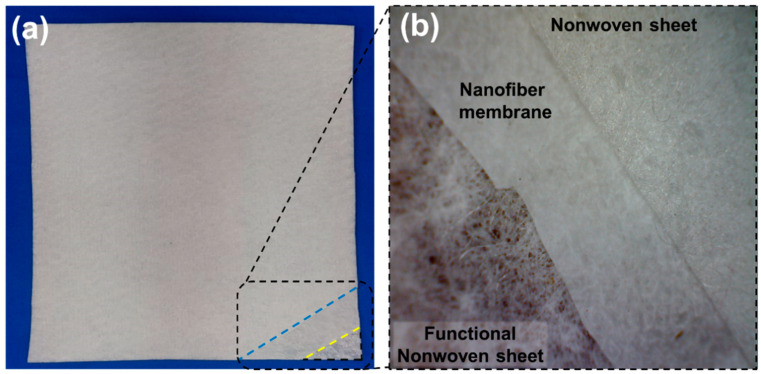
(**a**) Photographic view of nanofiber membrane-based nonwoven sweat pad, (**b**) Photographic view of three layers in nanofiber membrane-based nonwoven sweat pad.

Figure 7a shows the photographic view of the nanofiber membrane-based nonwoven sweat pad, and Figure 7b shows the photographic view of three layers in the nanofiber membrane-based nonwoven sweat pad. The developed nonwoven layers-based sweat pad is evaluated and compared through the thermophysiological characteristics. Different comfort characteristics such as air permeability, thermal resistance, moisture management, and water vapor permeability of these sweat pads are discussed.

**Figure 8:**
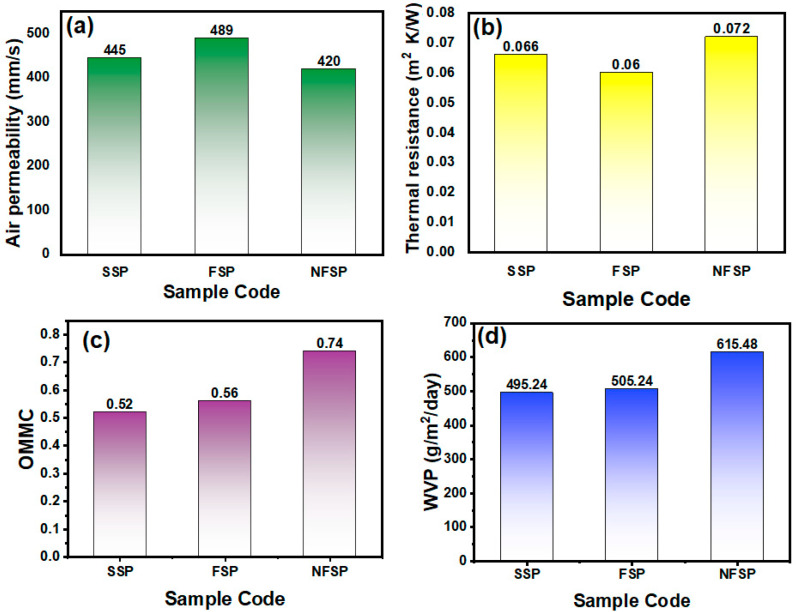
Comfort properties (**a**) Air permeability, (**b**) Thermal resistance, (**c**) OOMC, (**d**) WVP of the different sweat pad samples.

Figure 8a shows the air permeability of 445, 489, and 420 mm/s of sweat pad sample SSP, FSP, and NFSP, respectively. The NFSP sweat pad is slightly less air permeable; the presence of a nanofiber membrane between the nonwoven layers lowers the porosity, which merely affects the air permeability. However, all the samples have good air permeability and can effectively work for the sweat pad application. Figure 8b shows the thermal resistance of the different sweat pad samples SSP, FSP, and NFSP. The NFSP sweat pad shows thermal resistance of 0.072 m^2^ K/W, whereas SSP and FSP exhibit thermal resistance of 0.066 and 0.060 m^2^ K/W, respectively. The sweat pad with nanofiber membrane has a slightly higher heat resistance compared to other nonwoven samples.

The moisture management of the nonwoven layers was evaluated by the overall moisture management (OMMC) of samples. The OMMC represents the ability of the designed nanofiber-based sweat pad to transfer moisture from the inner surface of the sweat pad to the outer surface. Moisture management is the key characteristic for the sweat pad to estimate its performance, as it depicts the absorption, transportation, and evaporation of sweat from the body using this functional sweat pad. Figure 8c demonstrates the OMMC of the developed sweat pad SSP, FSP, and NFSP. The SSP and FSP samples show OMMC values of 0.52 and 0.56, respectively, whereas NFSP has an OMMC of 0.74. The sample NFSP has higher OMMC compared to SSP and FSP; the nanofiber membrane in the NFSP sample offers a higher surface area and absorption due to the higher surface area. It was also previously reported that PA6 nanofiber with a finer diameter shows higher water uptake compared to nanofiber with a coarser diameter [37]. Similarly, another study of blended nanofiber membranes also revealed that nanofiber webs, composed of nanofiber having finer diameters, showed an increase in water absorption [38]. The smaller diameter attributed to the large pore size resulted in higher moisture absorption. So, finer fibers lead to higher surface area and improve moisture absorption. The microporous structure of the nanofiber membrane in the NFSP sample provides the effective evaporation of the moisture from the sweat pad. Figure 8d shows the WVP of the three different sweat pad samples, SSP, FSP, and NFSP, at a WVP of 495, 505, and 616 g/m^2^/day. The NFSP sweat pad comprises a nanofiber membrane, which attributes to the higher water vapor permeability as compared to other sweat pads. These results are favorable in terms of avoiding sweat visibility on the wearer’s clothes. Comparing these properties, it is noted that the NFSP sweat pad shows significantly better comfort properties than the SSP and FSP samples.

## 4. Conclusions

This study aims to develop a more functional and breathable sweat pad with antimicrobial and quick drying performance. Three layered functional sweat pads (FSP) are designed in which the inner layer is made of an optimized needle-punched coolmax/polypropylene nonwoven blend. This layer is then dipped in antimicrobial ZnO solution (2, 4, and 6 wt.%), and super absorbent polymer (SAP) is embedded. Electrospun nanofibers of polyamide-6 are optimized for bead-free fibers. They are used as the middle layer to enhance the pad’s functionality, and the third layer is again made of needle-punched optimized coolmax/polypropylene nonwoven sheets. Nonwoven sheets are optimized based on better comfort properties, including air/water vapor permeability and MMT. It was observed that the multichannel structured coolmax shows more air permeability (882 mm/s). Whereas moisture transformation from the inner to outer layer through optimized electrospun nanofibers was better than other samples due to the ability to transfer moisture from the inner to the outer layer. The antimicrobial activity of the functional nonwoven layer due to the presence of ZnO nanoparticles was much better than SSP and FSP against *Staphylococcus aureus*. This study demonstrated that the nonwoven web blends of different fibers in the first layer provide better moisture management properties for advanced functional pads and also enhanced antibacterial properties by the addition of ZnO.

Further studies can be conducted to optimize the antimicrobial activity of other metal oxides in functional pads. The nanoweb of metal oxides can also be fabricated in sweatpants.

## Figures and Tables

**Table 1:** Optimization of electrospun nanofibers of PA-6.

Sample Code	Concentration (%)	Voltage (kV)	Flow Rate (µL/h)
NF1	18	30	150
NF2	18	28	200
NF3	18	32	200
NF4	16	32	250
NF5	16	28	150
NF6	18	30	250
NF7	14	30	250
NF8	14	32	200
NF9	16	30	200
NF10	14	28	200
NF11	16	30	200
**NF12**	**14**	**30**	**150**
NF13	16	28	250
NF14	16	32	150
NF15	16	30	200

**Table 2:** Comfort characteristics of the optimized PA6 nanofiber membrane.

Sr	Diameter (nm)	Porosity (%)	Air Permeability (mm/s)	Thermal Resistance (m^2^ K/W)	OMMC	WVP (g/m^2^/Day)
Optimized NF12	180 ± 29	62.17	398	0.062	0.85	1065

**Table 3:** Optimization of nonwoven web samples with coolmax and polypropylene.

Sample Code	Coolmax (%)	Polypropylene (%)
**NW1**	**100**	**0**
NW2	90	10
NW3	75	25
NW4	60	40
NW5	50	50
NW6	40	60
NW7	25	75
NW8	10	90
NW9	0	100

**Table 4:** Comfort characteristics of the optimized nonwoven layer.

**Sr**	**Air Permeability** **(mm/s)**	**Thermal Resistance** **(m^2^ K/W)**	**OMMC**	**WVP** **(g/m^2^/Day)**
Nonwoven sample (NW1)	398	0.062	0.85	1065

**Table 5:** Comfort characteristics of the optimized functional nonwoven layer.

Sr	Air Permeability (mm/s)	Thermal Resistance (m^2^ K/W)	OMMC	WVP (g/m^2^/Day)
Nonwoven sample (NW1)	719	0.041	0.74	719.38

## Data Availability

Data will be provided on request.
